# Evaluation of the Adhesion Strength of Ultrathin Gold Coatings on Substrates of Soda-Lime Glass and Cyclo-Olefin-Polymer by Cross-Cut and Scratch Tests under the Influence of a Thermal Shock Test for Use in Biosensors

**DOI:** 10.1021/acsomega.4c08288

**Published:** 2025-01-09

**Authors:** Rebecca Vornweg, Mona Roser, Alexander Fromm, Frank Burmeister, Thomas Günther, André Zimmermann

**Affiliations:** † Institute for Micro Integration (IFM), University of Stuttgart, Allmandring 9B, 70569 Stuttgart, Germany; ‡ Fraunhofer Institute for Mechanics of Materials (IWM), Woehlerstrasse 11, 79108 Freiburg, Germany; § Hahn-Schickard, Allmandring 9B, 70569 Stuttgart, Germany

## Abstract

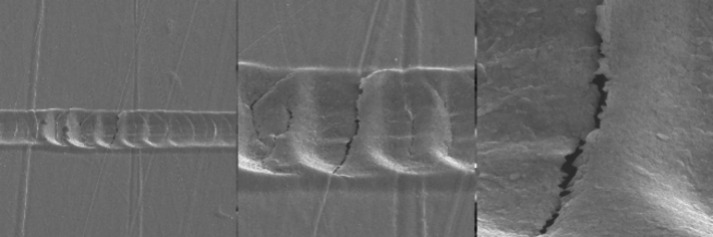

The current demand for highly sensitive, optical sensors in biodiagnostics has prompted the development of ultrathin metal coatings on a range of substrates. Given the potential attenuation of the signal from a plasmonic sensor for the detection of fluorescent molecules when an adhesion layer between the substrate and coating is employed, this study examines the impact of various factors on the adhesion strength between gold coatings and substrates comprising glass and cyclo-olefin-polymer (COP). The objective is to identify potential configurations for high adhesion strength, thereby eliminating the need for an adhesion layer in the fabrication of optical sensors with gold coatings for diagnostic applications or to utilize a minimal adhesion layer thickness. The influence of pretreatments prior to coating deposition with oxygen plasma, ultrathin adhesion layers of titanium as well as the impact of thermal shock tests with various cycles on the coating’s integrity are investigated. Two methods of adhesion strength testing were applied to ultrathin coatings on glass and polymer substrates. Cross-cut and nano scratch tests were used to assess the adhesion of the ultrathin metal layers, which provided qualitative and quantitative results. The results of the two test methods on glass substrates are in good accordance, demonstrating the highest adhesion strength for gold coatings on glass with an adhesion layer, in combination with or without plasma treatment. The cross-cut testing on COP demonstrates a constant high adhesion strength when using an adhesion layer alone or without any pretreatment, while a low adhesion strength is observed in combination with plasma treatment. Following the thermal shock test, alterations in adhesion strength were observed for the remaining configurations. The rise in the coefficient of thermal expansion of COP between +30 and 85 °C in comparison to lower temperatures, as evidenced by thermomechanical analysis, may account for the disparate adhesion strengths of COP following thermal shock tests.

## Introduction

**Figure 1:**
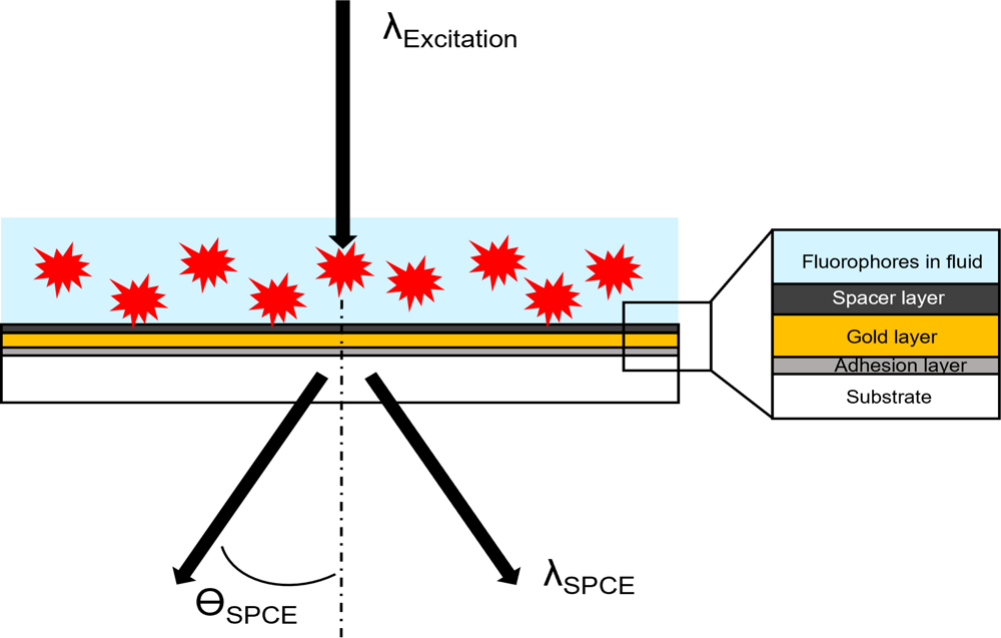
Schematic of an SPCE-sensor.

The high prevalence of diseases today has led to an increased demand for low-cost and time-efficient diagnostic tests that can be easily evaluated at the point of care, near the patient.^1,2^ As a result, research groups have been developing new diagnostic methods for decades that offer comparable sensitivity to existing methods in central laboratories and can be integrated into miniaturized setups at a low cost. One approach for detecting fluorescent molecules is through the plasmonic effect, using surface plasmon-coupled emission (SPCE). SPCE occurs when fluorescent molecules, for example the fluorescent labels on antibodies, excite oscillating electrons in a metallic layer, so-called plasmons. This results in the conversion of the isotropic fluorescent radiation into an anisotropic, wavelength-dependent plasmonic radiation, which can be read out and used to quantify the fluorescent molecules.^3,4^ For SPCE, an ultrathin metal coating of mostly gold, generally ranging from 30 to 100 nm, is applied as the amplifying sensor layer on various substrate materials. Gold surfaces are frequently employed in biomedical applications due to their favorable protein adhesion properties, see the work of Abad et al.^5^ Furthermore, the work of Reznickova et al.^6^ demonstrated enhanced cellular proliferation of vascular smooth muscle cells on sputtered gold layers. In general, sputtered layers such as those employed in this study are of an exceedingly high purity.^7^ Given that pure gold is regarded as biocompatible sputtered gold layers can be used and investigated in the initial phase of the process as a biocompatible surface for biomedical applications.^8^ For SPCE sensors in reflection, the selection of substrate materials is more flexible than for transmission setups, which require transparent materials such as glass^9^ or amorphous polymers.^10^ It is advised that the use of adhesion layers is avoided or minimized in order to guarantee optimal signal transmission without any loss of sensor signal quality.^11^Figure 1 illustrates the schematic structure of a SPCE-biosensor in a transmission setup. The use of polymer substrates allows lower costs for manufacturing and a better scalability in comparison with glass through the application of injection molding or hot embossing.^10,12^ COP is a commonly used substrate material due to its low autofluorescence, high optical transparency and low water absorption.^13^ In commercial point-of-care applications, the sensors may be exposed to environmental stresses, such as temperature fluctuations and humidity differences. Hence, it is imperative to examine the adhesion strength of those ultrathin gold coatings on various substrate materials under different environmental conditions.

The adhesion strength is significantly affected by the difference in thermal expansion between gold and substrate material. For instance, soda-lime glass^14^ has a thermal expansion coefficient of 9.0 × 10^–6^ K^–1^ and polyimide^15^ 20 × 10^–6^ K^–1^, while the most commonly used value of gold as bulk^15^ material is 14.2 × 10^–6^ K^–1^. In the research conducted by Oliva et al.,^15^ it was observed that the thermal expansion of gold layers with a thickness of 10 nm was approximately five to six times greater than that of the bulk material. Additionally, it is worth noting that noble metals, including gold, do not adhere well to nonmetallic materials such as silicate glasses or polymers. This often necessitates the use of an adhesion layer and pretreatment steps.^16−18^ The low chemical reactivity and low affinity of noble metals such as gold to oxygen contribute to their poor adhesion to nonmetallic substrates.^19,20^ Moreover, substrates such as glasses or polymers lack free oxygen bindings on their surface.^21^ The low adhesion strength between polymers and metals is also due to the contrasting properties of the two materials. Metals exhibit closely packed crystalline structures, while polymers have large covalently bonded macromolecules with very weak van der Waals interactions and a cohesive energy that is usually two magnitudes lower than that of metals.^22,23^ Various methods have been investigated in recent decades to increase the adhesion strength. One approach is to roughen the surface through plasma treatments to increase mechanical interlocking. Nevertheless, this method is not advised for use with optical devices, as the presence of roughness increases the amount of scattered light, thereby reducing the signal-to-noise ratio. Other approaches promote the chemical bonding between the substrate and the coating. Plasma treatment can be applied to glass to remove residuals and break the Si–O–Si bonds into Si–O covalent bonds, creating free oxygen radicals. This supports the binding of metals with a high affinity to oxygen, such as titanium or chromium, which are typically used as adhesion layers.^19,21^ The utilization of adhesion layers is a common practice employed to enhance the adhesion strength. The research conducted by Li et al.^22^ illustrates that plasma treatment in conjunction with an adhesion promoting layer, here Al_2_O_3_/Al, can enhance the adhesion strength between a gold coating and a polysiloxane-based polymer. For some material combinations, an alloy is formed between the coating and the adhesion layer, as evidenced by the research conducted by Todeschini et al.^19^ The formation of an alloy between chromium and gold was observed as a further chemical bonding mechanism, which resulted in enhanced mechanical stability when compared to titanium, where no alloy was formed. However, further studies have investigated the potential negative impact of adhesion layers on the SPCE signal in the context of their application in optical sensors. A 2 nm thick adhesion layer of titanium was employed by Habteyes et al.,^11^ which resulted in a damped SPCE signal. This finding is also reported in the work of Lahiri and Colas et al.^16,24^ Abbott et al.^25^ investigated the adhesion properties of various materials, including titanium, chromium, and tungsten, with a thickness of either 0.5 and 2 nm. Their findings revealed that titanium exhibited superior adhesion strength compared to tungsten and chromium, but for SPCE signals tungsten demonstrated enhanced performance. Consequently, achieving high adhesion strengths without an adhesion layer between a thin gold coating and a nonmetallic substrate represents a significant challenge. Therefore, specialized measurement methods that take additional, application induced stresses into account, are required. Despite the extensive research on thin films in sensor systems, there is still a lack of general standards for testing adhesion strengths of ultrathin coatings. The current methods for measuring adhesion strength include the cross-cut method, pull test, scratch test, and the Rockwell indentation test.^26,27^ One drawback is their lack of comparability and their limited applicability toward ultrathin ductile coatings. The indentation test is recommended for hard coatings, especially for ceramic coatings, and not recommended for elastic coatings on hard base materials.^28,29^ The cross-cut test is applicable to hard substrates with a thickness of more than 0.25 mm and soft materials (>10 mm).^30^ The standardized scratch test protocol describes the characteristics of ceramic coatings, but does not specify the same for other materials.^31^ In the pull-off test the stamp of a tensile testing machine is applied to the coating surface using an adhesive. In the case of thin coatings, the adhesive can diffuse through the coating, potentially influencing the result of the adhesion strength.^32^ In general, it should be noted that it is challenging, if not impossible, to quantify adhesion strength in absolute terms. Instead, measurements are inherently comparative.^26^

In order to meet the needs of the mass market, the use of polymers is essential as replication speed at reduced costs can be addressed. For SPCE sensors, the use of adhesive layers should be avoided or their thickness minimized to optimize the signal quality, since the adhesion layers contribute heavily to the losses. The use of gold is essential due to its biocompatibility. The key question is whether this combination can be realized effectively, especially under the required temperature conditions for future applications, and whether adhesion strengths comparable to those on glass are possible on polymer substrates. The influence of pretreatments with oxygen plasma prior to coating deposition, adhesion layers of titanium as well as the impact of thermal shock tests with various cycles on the coating’s integrity are investigated. In order to respond to these questions, it was necessary to determine which measurement method would be appropriate for evaluating adhesion strength. Given the difficulty in measuring the adhesion strength of thin films, a variety of coatings were produced and tested using different methods. The objective is to identify a method that would provide reliable results. Two methods for testing the adhesion strength are applied: the cross-cut test, which is a fast and qualitative method, and the scratch test, which is a more quantitative method with less handling impact and has been shown to be suitable for thin films. The cross-cut test is specified in the DIN standard DIN EN ISO 2409.^30^ However, the standard lacks details regarding the procedure and the equipment. The tool which is used to cut a cross through the coating into the substrate is not defined, nor is the tape specified that should be used to remove loose particles after the test. Six cross-cut values are defined through the images, which illustrate the removal of coating material at varying percentages. The value 0 describes the complete preservation of the layer and 5 the complete detachment of the layer. In comparison, the scratch test is a more automated testing method that provides both, qualitative and quantitatively comparable results. In particular, the Nano Scratch Tester NST^3^ used is well-suited for the analysis of thin layers with a thickness of less than 1000 nm and has not been widely investigated for ultrathin gold coatings on various substrate materials. Quantitative results are obtained when the images are combined with force–displacement diagrams.^33^

## Materials and Methods

Soda-lime glass microscope slides with a surface roughness *R*_a_ of 5 nm and injection molded COP substrates with *R*_a_ of 16 nm were used as samples. COP 690R was chosen due to its high chemical stability, which allows for lithographic processing, and its optical transmission properties making it suitable as an economic substitute to glass, in particular regarding its machinability. The glass substrates have a thickness of 1 mm, while the COP substrate has a thickness of 1.5 mm. Before processing, the substrates were cleaned for 5 min in an isopropanol solution using an ultrasonic device, rinsed with distilled water and dried with nitrogen. Following the cleaning process, the substrates were coated with gold using a sputter coater Creavac CREAMET (750 Cl2). Four different configurations of the samples were investigated: The first set of samples was directly coated with 50 nm of gold, the second set was treated by oxygen plasma before coating. The third configuration was without oxygen plasma and with an adhesion layer of 0.5 nm titanium while the fourth configuration included oxygen plasma and an adhesion layer. The plasma treatment was carried out in the load-lock chamber of the PVD system at a pressure of 5 × 10^–2^ mbar for only 1 min to prevent roughening by etching as recommended in the work of Shenton et al. and Shinohara et al.^34,35^ The distance between the samples and the electrode was 95 mm and the electrode power was 600 W. Gold was sputtered on a magnetron target at 500 W with a deposition rate of 22 nm/min and titanium on a DC target at 300 W with a deposition rate of 4 nm/min, each target being four inches in size. The pressure during sputtering was kept at 6 × 10^–4^ mbar. Titanium as adhesion layer was chosen due to its low diffusion, stability over time and its compatibility with lift-off techniques.^19^ It was applied with a thickness of approximately 0.5 nm. The coating thickness for the adhesion layer was selected based on two criteria: the results of our simulations and the findings of previous studies by other authors.^11,16,24^ The simulations demonstrated that the sensor signal exhibited the deepest and sharpest resonance in the absence of titanium. As the layer thickness increases, the resonance becomes less sharp, which subsequently reduces the sensor signal. Consequently, if titanium is employed, it should be coated as thinly as possible with the PVD system. The results of the simulations are in accordance with the findings presented by the aforementioned authors. However, the simulations are not presented in this paper to avoid overcomplicating the discussion.

To further investigate the adhesion strength, a two-chamber thermal shock test was performed with a maximum temperature of +85 °C and a minimum temperature of −40 °C. Each temperature level was held for 15 min to obtain a uniform temperature of the samples. 50, 500, and 1000 cycles were carried out.

**Figure 2:**
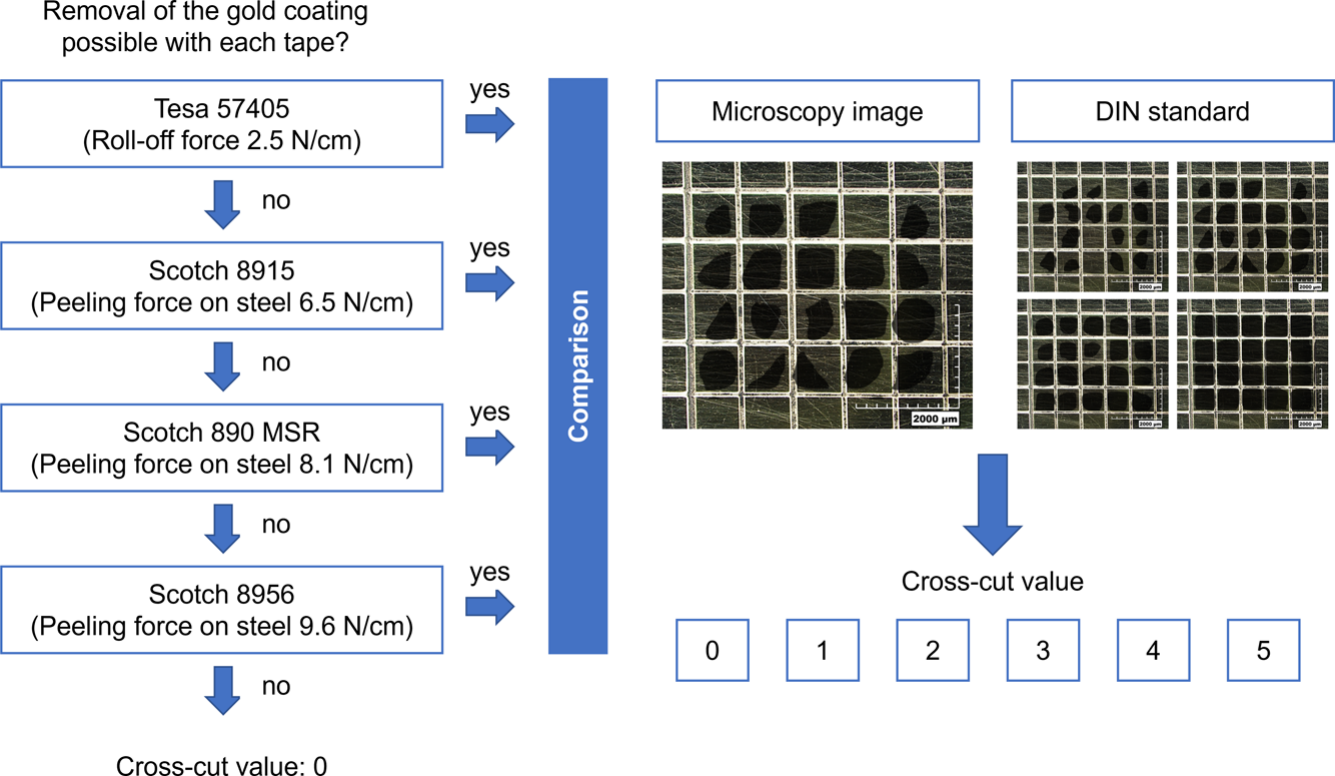
Procedure of the cross-cut method.

The results of the scratch test were compared with those of a standard cross-cut test. The cross-cut test was performed on three samples of the same parameter set on a single occasion. As stated in the DIN EN ISO 2409, any loose particles were removed with an adhesive tape. In order to increase the quantitative significance of this test, four tapes with different adhesive strengths on steel from 2.5 to 9.6 N/cm were used. The procedure is shown in Figure 2. Following the cross-cut tests, each sample was evaluated according to the DIN EN ISO 2409. Thereafter, adhesive tape with a higher adhesion strength was only used if the weaker adhesive tape did not lead to any delamination. The mean value in N/cm was calculated based on the adhesion strength of the tape and the percentage of the lifted material of each cross-sectional characteristic value.

**Figure 3:**
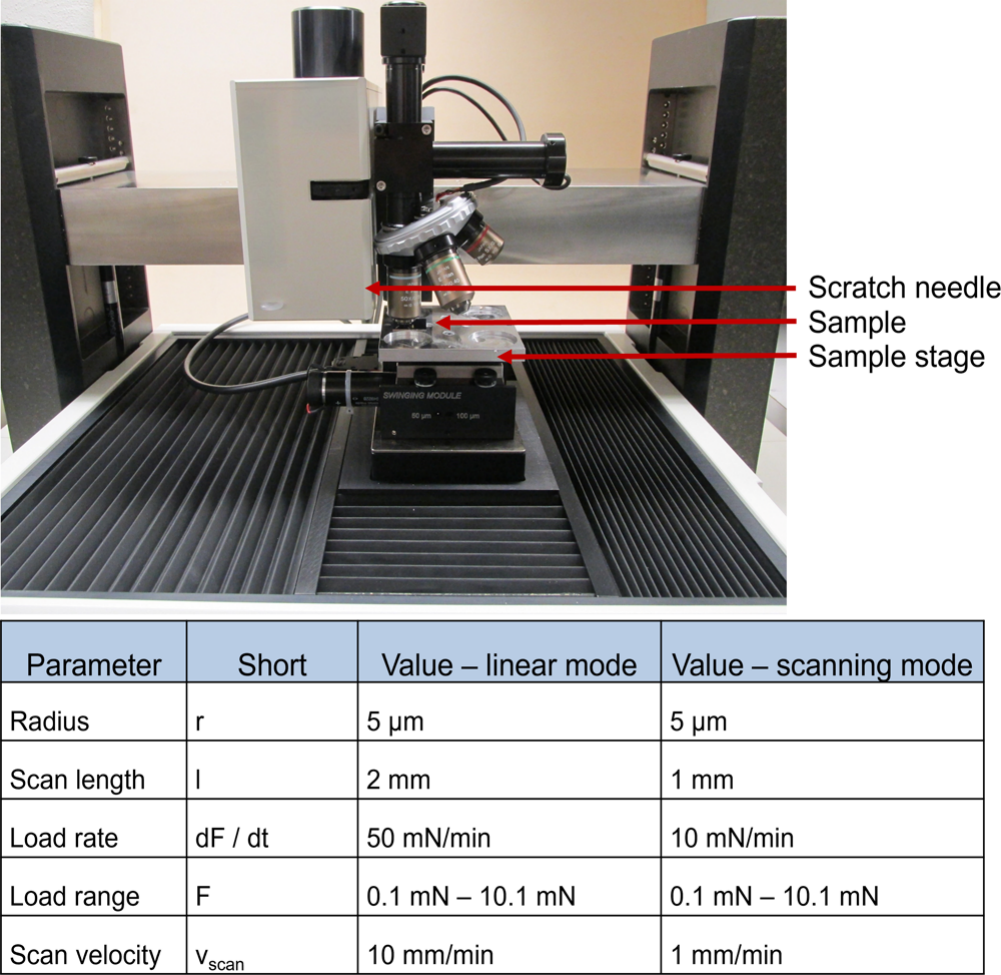
Measurement setup and final parameters of the scratch test.

The Nano Scratch Tester NST^3^ from Anton Paar Germany GmbH was employed for the scratch tests. For each parameter set, a single sample was manufactured and subjected to investigation, with three scratches made on each sample and subsequently examined using digital microscopy images, SEM (scanning electron microscopy) and force–displacement diagrams. The scratches were generated with a microneedle that traveled across the surface of the sample with a linearly increasing normal load. The critical load, defined as the force at the onset of coating failure, is determined by the combined effect of the elasto-plastic stresses induced by the inserted needle, the frictional stress and the residual stress of the coating.^26^ At the beginning of the tests, two different diamond needles, with radii of 5 and 50 μm, with different scratch parameters, were employed. However, the smaller tip radius delivered the more reliable and reproducible results and was exclusively used for the final tests. In addition to the linear scratch mode, a so-called scanning scratch mode was also implemented, a custom-built feature of the Fh-IWM scratch tester. There, an oscillating motion perpendicular to the linear scratch direction, is superimposed on the motion of the scratch needle. This sometimes enhances the discrimination capability of the test and proved successful especially for the gold coatings on glass. Prior to each scratch, a prescan with a constant low force (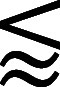 0.5 mN) was carried out, similar to a profilometer. The measured surface topography was taken into account for the consecutive measurements with higher forces and used to compensate for geometric effects, substrate unevenness and tilting. Figure 3 illustrates the measurement setup and the final parameters used for the scratch testing.

## Results

**Figure 4:**
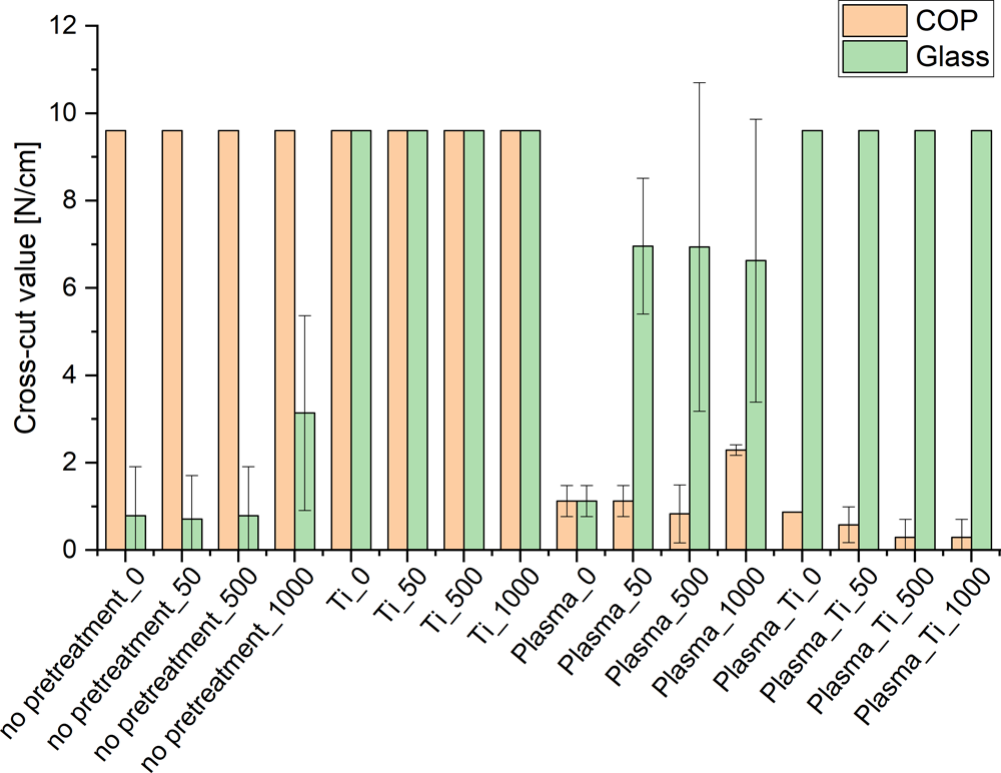
Results of cross-cut test on glass and polymer substrates.

Figure 4 illustrates the outcomes of the cross-cut tests on glass and COP substrates. The diagram depicts the calculated cross-cut values as a mean value and with standard deviation for each parameter set. With the exception of the adhesion values of the untreated and plasma-treated glass substrates, the evaluated adhesion values exhibit minimal variation.

Preliminary investigations have demonstrated that the nano scratch test is unsuitable for utilization on COP substrates, as no reproducible outcomes can be attained from samples with a ductile coating on a soft substrate. Therefore, initial scratch tests were conducted on glass substrates to compare the applicability of the two methods: linear and scanning method. The linear method was found to be less appropriate, with only two of 12 samples exhibiting layer detachment. Consequently, further tests were conducted using the scanning method, which employs a superimposed oscillating movement of the needle with a frequency of 15–30 Hz and an amplitude of 50 μm. This increased the sensitivity of the measurement. In comparison to the linear method, the scratch length was reduced by half, to 1 mm, and the load rate was doubled to 10 mN/min. This enabled more quantitative results and showed layer detachment for 6 samples. The results of the evaluated critical load are summarized in Table 1. The standard deviation was sufficiently low, thereby demonstrating the method’s capacity to provide valid and repeatable values.

**Table 1:** Comparison of Results of Linear and Scanning Method of Scratch Test on Glass Substrates

sample	linear method [mN]	scanning method [mN]	damage pattern [-]
no pretreatment_50	4.24 ± 0.25	1.63 ± 0.13	3
Ti_50	-	-	1
Plasma_50	-	4.74 ± 0.34	3
Plasma_Ti_50	-	-	1
no pretreatment_500	4.42 ± 0.36	1.69 ± 0.10	3
Ti_500	-	-	1
Plasma_500	-	8.64 ± 0.04	2
Plasma_Ti_500	-	-	1
no pretreatment_1000	-	8.40 ± 1.07	2
Ti_1000	-	-	1
Plasma_1000	-	8.45 ± 0.12	2
Plasma_Ti_1000	-	-	1

## Discussion

In order to investigate the adhesion strengths for different configurations under the temperature conditions required for future applications and to determine the suitability of the scratch method for thin ductile coatings, such as gold, on hard substrate materials, the test results are discussed below.

### Influence of Pretreatment and Adhesion Layer

**Figure 5:**
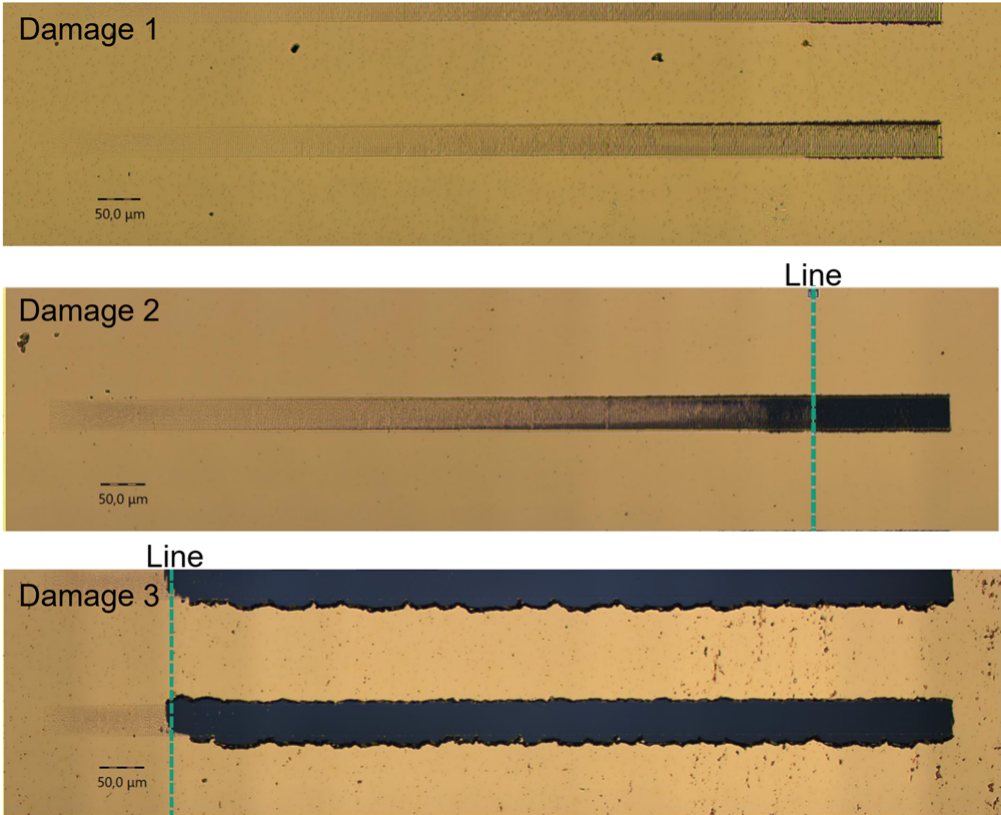
Different types of damage pattern of the coatings; on the top damage pattern 1: samples with adhesion layer; in the middle damage pattern 2: samples without adhesion layer; on the bottom damage pattern 3: samples without adhesion layer.

Following the completion of the scratch tests, light microscope and SEM images of the scratch and the associated force–displacement diagrams were subjected to analysis to identify any distinctive features, such as cracks or spallations. Due to the thinness of the coatings, only minor or no dips in the force–displacement diagram could be observed. However, in conjunction with the optical images, it was possible to reproducibly define a critical load. The results of the scratch tests are depicted in Table 1. The optical analysis showed three different types of damage as illustrated in Figure 5. In damage pattern 1, no layer detachment could be observed, which precluded the determination of a critical load. Damage pattern 1 could be observed on all samples with an adhesion layer. No detachment of the gold coating could be detected, and there was no difference between samples with or without plasma pretreatment. In the absence of titanium, the gold coating was observed to detach, resulting in the manifestation of damage patterns 2 and 3. Damage pattern 3 shows a detachment at lower critical loads, with this detachment occurring with greater suddenness compared to damage pattern 2. It was thus possible to ascertain the critical load, defined as the onset of a “gold-free” area in the scratch. The critical load is indicated in Figure 5 by vertical lines.

**Figure 6:**
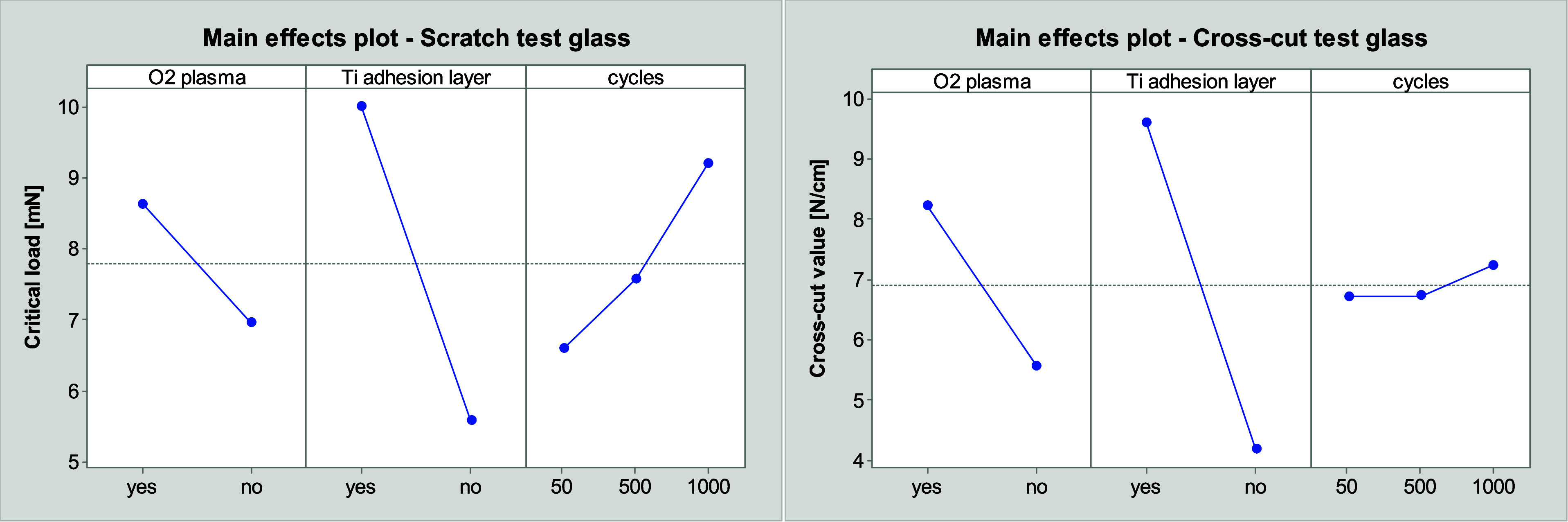
Main effect plots of scratch test on glass (left) and cross-cut test on glass (right) with cross-cut values related to the adhesive strength of the adhesive tape, plots derived from DOE using “Minitab”.

The main effect plots in Figure 6, which were derived from a Design of Experiments (DOE) conducted with the software “Minitab”, illustrate the influence of the different parameters on the adhesion strength. In order to facilitate comparison between the results of both methods, the cross-cut values are converted into a factor of the cross-cut value of the detachment in percentage and the respective adhesive force of the tape in N/cm. It can be concluded that the highest adhesion strength is achieved with a cross-cut value of 9.6, provided that the layer does not peel off when the strongest adhesive tape is utilized. The results of both methods demonstrate a similar trend. Nevertheless, the influence of cycles of the thermal shock test is less discernible in the cross-cut test than in the scratch test.

The increase in adhesion strength when using an adhesion layer is consistent with previous research,^19,22^ and confirms the hypothesis of improved binding between gold and glass surfaces when using titanium as an adhesion layer. In addition, the application of a plasma treatment prior to the coating of gold resulted in an enhanced adhesion strength, in line with the expected result.

**Figure 7:**
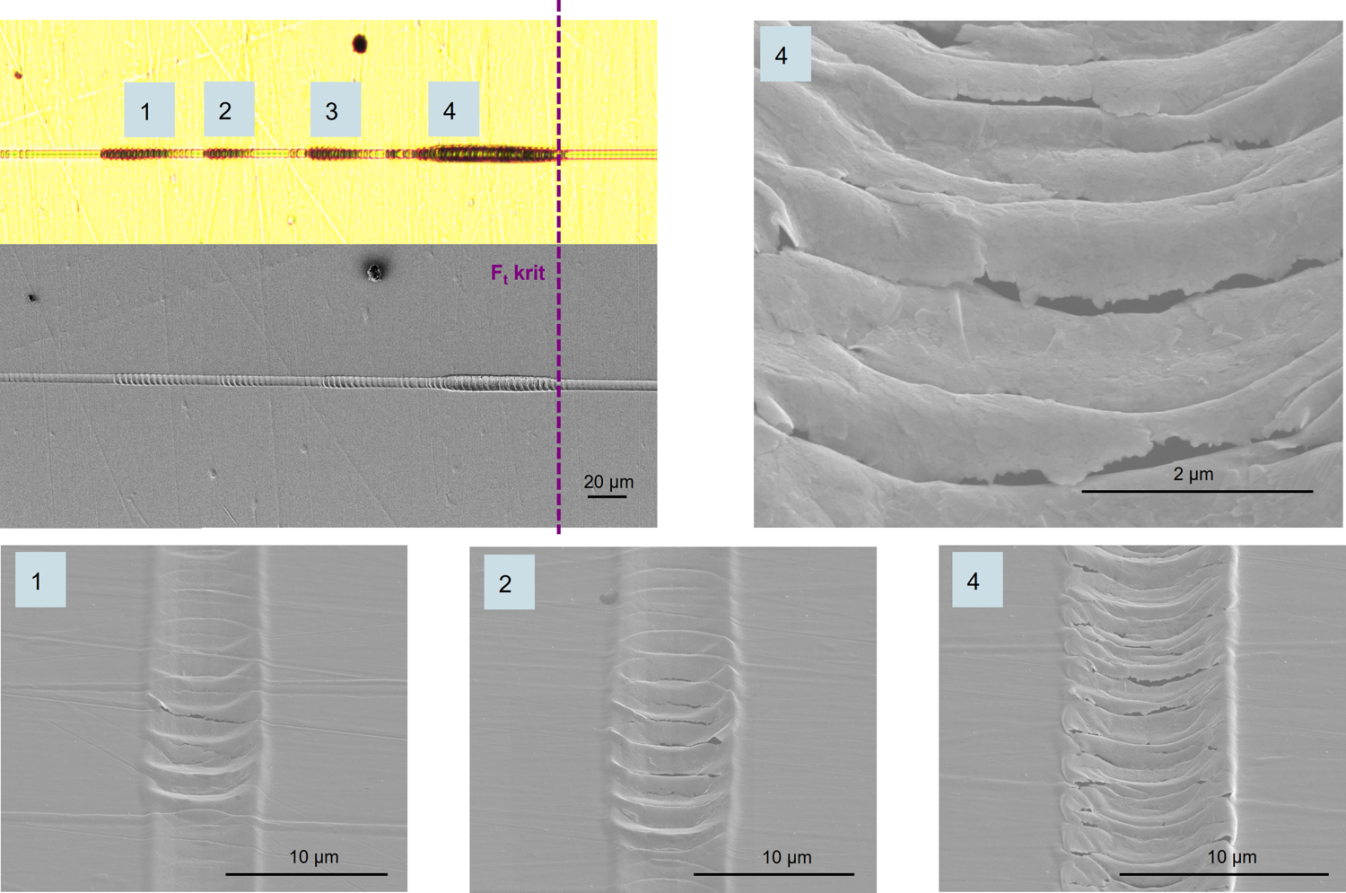
Microscopy and SEM images of a scratch on 50 nm thick gold coating on COP substrates.

The transferability of the scratch test method for gold coatings on substrate materials of lower hardness than glass is not straightforward. The main reason is the significant elastic and plastic deformation of the substrate as a consequence of the impact of the needle, which in turn results in a significantly deformed surface and a completely different strain and stress distribution in the vicinity of the scratch stylus. Furthermore, the high ductility of the gold layer leads to deformations of the layer rather than its detachment. Figure 7 illustrates a scratch on a COP substrate with a 50 nm thick coating. The ripples are indicative of initial coating failure, but SEM examinations were necessary to identify the detailed failure mechanism. An initial crack in the gold coating is observed in ripple 1 and a continuous crack in the gold layer across the entire width of the scratch is evident in ripple 2 (Figure 7). The extent of the cracks increases from ripple 1 to ripple 4, and the crack opening increases. The scratch tip performs a stick–slip-like motion where the frictional force exerted by the tip (the lateral force signal is recorded but not shown here) induces a shear stress on the coating-substrate interface. At some critical point, the imposed stresses become bigger than the strength of the interface and delamination and crack formation sets in. In order to attempt an assessment, ripple 4 was tentatively defined as the critical load due to the high number of cracks and the strong detachment of the gold layer. However, it was not possible to establish a meaningful and consistent interpretation of the observed coating failures within the set of examined Au/COP samples.

**Figure 8:**
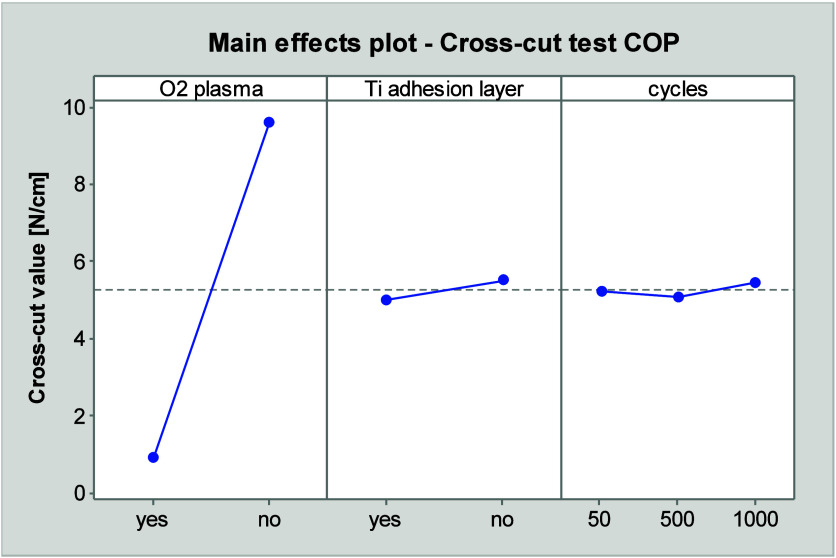
Main effect plot of cross-cut test on COP substrate with cross-cut values related to the adhesive strength of the adhesive tape, plot derived from DOE using “Minitab”.

The cross-cut results are presented in Figure 8, given the high degree of reproducibility that was achieved during the tests. Figure 8 illustrates the main effects that influence the adhesion strength of the COP substrates. It demonstrates that the adhesion strength is not increased with the use of oxygen plasma and a titanium layer. Additionally, a marginal increase in adhesion strength due to thermal shock is evident.

The lowest adhesion strength is observed in the case of plasma treatment in conjunction with a titanium layer. Without a titanium layer, but with plasma treatment, the adhesion strength is slightly higher. The remaining combinations exhibit higher adhesion strengths. In comparison, the results demonstrate a constant high adhesion strength when using an adhesion layer alone or without any pretreatment.

Plasma treatment on polymer substrates leads to cleaning, activation and cross-linking processes on the substrate. In the research done by Hegemann et al.,^36^ the various effects of plasma treatment on polymer substrates is shown. However, due to the inertness of gold and its lack of an oxygen binding site, the assumption of covalent bonding for improved adhesion does not apply. Another possibility can be the treatment time and power. According to Shenton et al.,^34^ reduced adhesion is caused by excessive treatment time resulting in molecular rotation, weak interfaces, and etching. It was found that oxygen plasma is less effective on polymer substrates compared to tetrafluoromethane with oxygen.^22^ Further testing is required to gain a deeper understanding.

### Influence of the Number of Cycles of the Thermal Shock Test

In comparison to pattern 2 on glass, pattern 3 exhibits a remarkedly reduced adhesion strength, with the detachment of the gold layer occurring with greater abruptness. This pattern occurs only for low numbers of thermal cycles. Pattern 2 is observed after the application of a larger number of temperature cycles. This pattern is evident in samples subjected to 500 and 1000 cycles. This suggests that an increase in adhesion strength is accompanied by an increase in the number of cycles.

**Figure 9:**
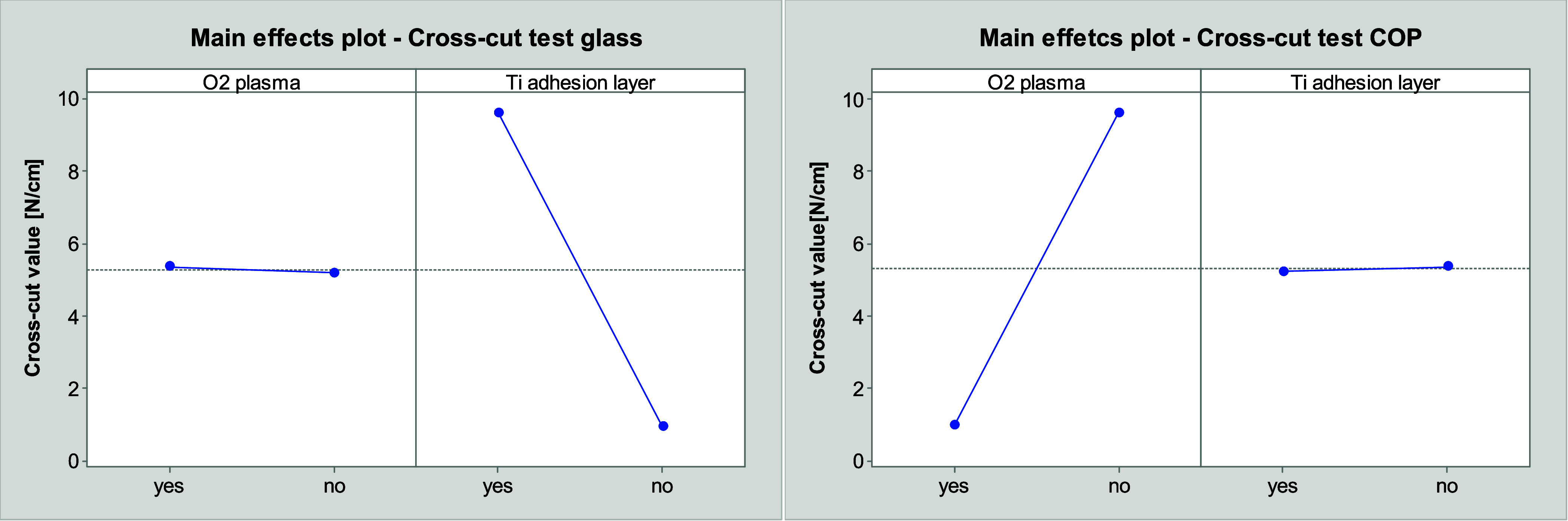
Cross-cut values of glass (left) and COP (right) substrates without applied thermal shock test with cross-cut values related to the adhesive strength of the adhesive tape, plots derived from DOE using “Minitab”.

A comparison of the cross-cut values of the substrates with 0 cycles, which means without the application of a thermal shock test, demonstrates a discrepancy. In the case of glass substrates, the oxygen plasma treatment exerts only a low influence on the adhesion strength, as evidenced by a comparison of Figures 9 (left) and 6. Samples that have not undergone plasma treatment and lack an adhesion layer exhibit a reduced adhesion strength relative to samples subjected to the thermal shock test. Following the thermal shock test, the cross-cut value is elevated, as illustrated in Figure 4. Moreover, it was observed that substrates that had undergone plasma treatment without the application of an adhesion layer exhibited an enhancement in adhesion strength following thermal shock. The adhesion strengths on COP substrates differ slightly between the samples with and without thermal shock testing, as illustrated in Figures 9 (right) and 8. The influence of plasma treatment in combination with an adhesion layer before thermal shock testing is slightly higher in comparison to the samples that underwent thermal shock testing. After thermal shock testing, the cross-cut test values of the samples that were plasma treated without an adhesion layer were slightly increased. In contrast, the samples without any pretreatment or with an adhesion layer showed a constant cross-cut value as illustrated in Figure 4.

**Figure 10:**
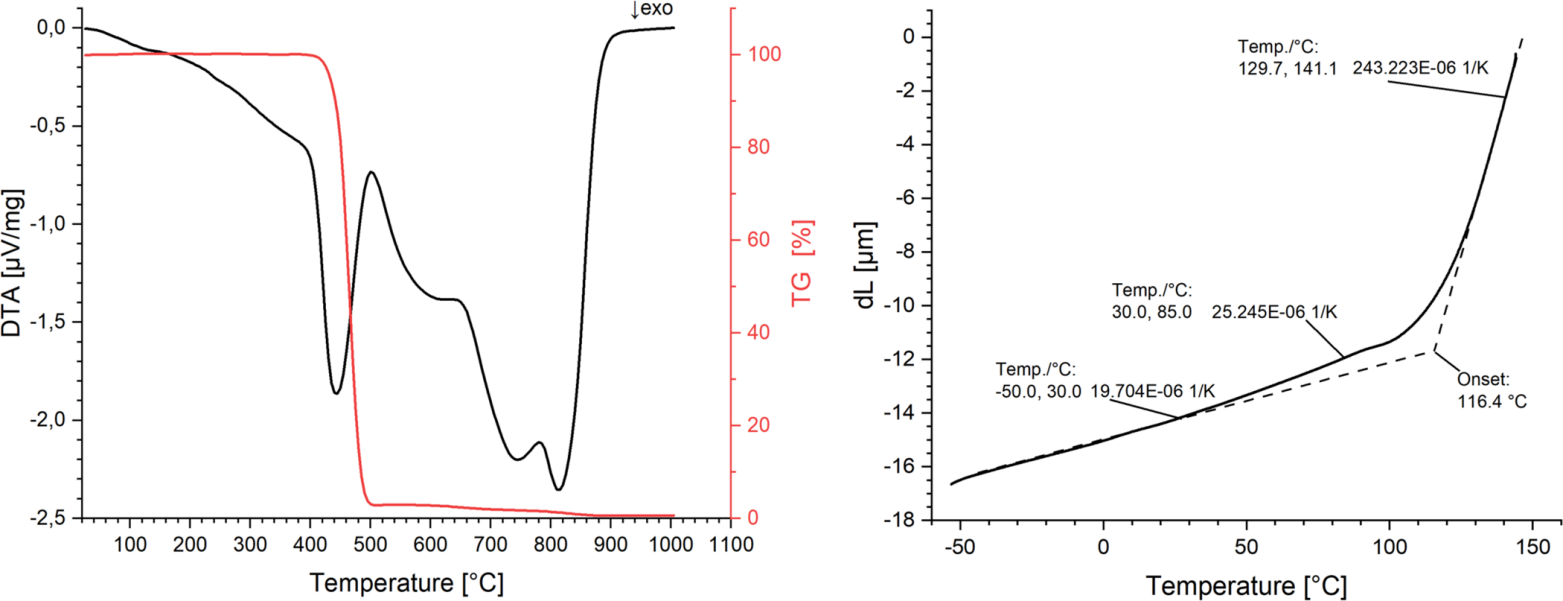
DTA-TGA (left) and TMA (right) of the injection molded COP substrate.

Thermal analyses were carried out due to the changing behavior of adhesion strengths on COP substrates after thermal shock. A thermomechanical analysis (TMA), a differential thermal analysis (DTA) and a thermogravimetric analysis (TGA) were each performed on a sample of the injection-molded polymer substrate. A combined DTA-TGA was conducted on the STA-409C apparatus, encompassing a temperature range of 25 to 1010 °C at a heating rate of 10 K/min. The results of the DTA show no exothermic or endothermic reaction between 25 and 85 °C, as shown in Figure 10 left. Only at 405 °C does the polymer substrate begin to degrade, which is in good agreement with the TGA results. The mass decreases and an exothermic reaction starts. The degradation ends at 505 °C when the mass loss becomes constant and the first exothermic reaction is over. The TMA, conducted with the NETZSCH TMA 202, indicates a coefficient of thermal expansion of 19.7 × 10^–6^ K^–1^ within the temperature range of −40 to +30 °C. Between +30 and 85 °C the sample exhibits a slightly increased coefficient of thermal expansion of 25.2 × 10^–6^ K^–1^, as shown in Figure 10 right. This increase of the coefficient suggests the presence of heat-induced stresses in the thermal shock chamber, which could potentially influence the adhesion strengths.

The increase in adhesion strength can also be attributed due to aging effects in the metal layers.^37,38^ Liston et al.^39^ describe the hypothesis that diffusion determines the bond strength of polymers. Diffusion and aging can be promoted by temperature influence.

In conclusion, it can be stated that the adhesion strengths on glass substrates are, on average, slightly higher than those on COP substrates. The findings of the investigation suggest that the gold coating on glass substrates exhibits slightly elevated adhesion strength. Nevertheless, additional experimentation is required to gain a deeper comprehension of this phenomenon. A number of models exist to explain the adhesion between different materials. Glass forms Si–O–Si bonds, which can undergo a chemical transformation into covalent Si–O bonds. This process generates free oxygen radicals, which can subsequently combine with metals that exhibit a high affinity for oxygen and are therefore uses as adhesion layers.^19,21^ The PVD coating process also exerts an influence on the adhesion of the coating. The coating, produced under high vacuum conditions, has the effect of reducing oxidation and, consequently, adhesion, due to the diminished formation of the metal’s oxide-hydrate layer.^40^ Furthermore, it can be observed that the enhancement of the adhesion strength can be attributed to the elevated surface free energy (SFE) of the glass substrate and the significantly higher polar component in comparison to polymers. The SFE of soda-lime glass materials has been reported as 58.95 mJ/m^2^ by Güleç et al.^41^ and as 55.8 mN/m by Vicente et al.^42^ with a polar component of more than 60%. In comparison, polymer substrates such as COC, which is very similar to COP, have a lower SFE of between 32.8 and 39.7 mN/m, as reported by Shin et al.^43^ and offer a significantly lower polar component. Given that the surface energies of polymers and gold are comparable, with both materials exhibiting low surface free energies and low polar components, it can be posited that low-energy dispersive interactions are the primary mechanism responsible for the adhesion between polymers and gold.^44−46^ In addition, the low adhesion strength between polymers and metals is also influenced by the contrasting properties of the two materials. Metals have tightly packed crystalline structures, whereas polymers have large covalently bonded macromolecules with very weak van der Waals interactions and a cohesive energy typically 2 orders of magnitude lower than that of metals.^23^

## Conclusions

In conclusion, the influence of plasma treatment, adhesion layer of titanium and thermal shock testing on adhesion strength could be derived by employing the cross-cut and nano scratch test to investigate the adhesion strengths of gold coatings on glass and polymer substrates. The results of the cross-cut test demonstrated the efficacy of this method for qualitatively evaluating ultrathin gold coatings on glass and COP substrates. The scratch test is appropriate for thin ductile coatings, such as gold, when applied to hard substrate materials like glass; however, it is not suitable for the use on COP substrates. The results of the two test methods on glass substrates are in good accordance, demonstrating the highest adhesion strength for gold coatings on glass with an adhesion layer, in combination with or without plasma treatment. The samples without any pretreatment exhibited the lowest adhesion strength and are therefore unsuitable for the use as optical elements in diagnostic applications. An increase in adhesion strength is accompanied by an increase in the number of cycles of a thermal shock test. Samples that lack an adhesion layer demonstrate enhanced adhesion strength following the temperature shock test. In conclusion, the coating system on the glass substrate provides high and constant adhesion strength both before and after thermal shock when an ultrathin adhesion layer is applied. The cross-cut test shows that comparable adhesion strengths can be achieved on COP substrates in the absence of an adhesion layer, thereby preventing any potential deterioration of the sensor signal. High adhesion strength between the gold coating and the COP substrate can be achieved without an adhesion layer and without the plasma treatment. However, under thermal influence, the cross-cut test values of the samples that underwent plasma treatment without an adhesion layer were slightly increased. In contrast, the samples that were plasma treated and exhibited an adhesion layer displayed a decline in the cross-cut value. In general, the most constant values of the adhesion strength were obtained for the samples without any pretreatment or with a thin adhesion layer. The rise in the coefficient of thermal expansion of COP between +30 and 85 °C in comparison to lower temperatures, as evidenced by thermomechanical analysis (TMA), may account for the disparate adhesion strengths of COP following thermal shock tests. A combined differential thermal analysis (DTA) and a thermogravimetric analysis (TGA) of the COP substrate revealed no exothermic or endothermic reaction between 20 and 85 °C. Further studies should be conducted to investigate the effects of different parameters in PVD coating on coating adhesion. Additionally, it would be beneficial to examine further pretreatment methods and to perform a biotoxicity test of the coating system.
